# Synthesis of Bisindolylmethanes and Their Cytotoxicity Properties

**DOI:** 10.3390/ijms14011843

**Published:** 2013-01-16

**Authors:** Kalla Reddi Mohan Naidu, Shaik Ibrahim Khalivulla, Syed Rasheed, Sharida Fakurazi, Palanisamy Arulselvan, Ola Lasekan, Faridah Abas

**Affiliations:** 1 Department of Food Technology, Universiti Putra Malaysia, 43400 Serdang, Selangor, Malaysia; E-Mail: drrmnaidu@gmail.com; 2 Department of Chemistry, Yogananda Inistitute of Technology and Science, 517520 Tirupati, India; 3 Department of Pharmaceutical Chemistry, Faculty of Pharmaceutical Sciences, UCSI University, No.1, Jalan Menera Gading, 56000 Cheras, Kuala Lumpur, Malaysia; E-Mail: shaik@ucsi.edu.my; 4 Department of Chemistry, Sri Venkateswara University, 517502 Tirupati, India; E-Mail: rasheedsvu06@gmail.com; 5 Department of Human Anatomy, Faculty of Medicine and Health Sciences, Universiti Putra Malaysia, 43400 UPM Serdang, Selangor, Malaysia; E-Mail: sharida@medic.upm.edu.my; 6 Laboratory of Vaccines and Immunotherapeutics, Institute of Bioscience, Universiti Putra Malaysia, 43400 UPM Serdang, Selangor, Malaysia; E-Mail: arulbio@gmail.com; 7 Department of Food Science, Universiti Putra Malaysia, 43400 Serdang, Selangor, Malaysia; E-Mail: faridah@food.upm.edu.my

**Keywords:** cytotoxicity properties, bisindolylmethanes, dibisindolylmethanes, green catalyst

## Abstract

Polymer supported dichlorophosphate (PEG-OPOCl_2_) is an efficient green catalyst for the electrophilic substitution reaction of indole with aromatic aldehydes, in neat condition, to afford an excellent yield of bis(indolyl) methanes with short reaction time, at room temperature. The synthesized compounds and their anti-cancer activity are evaluated.

## 1. Introduction

Cancer has a significant social and economic impact on the human health care system and is one of the major causes of human mortality in the world. Almost half of the cancer incidence and mortality occurs in Asia, with lung and bronchus, breast, and colorectal cancers in women being the most common fatal cancers [1,2]. A major etiology of cancer and associated adverse effects is primarily from an unhealthy lifestyle and environmental pollution. Development of cancer cells from healthy cells involves mainly three important steps, namely initiation, promotion, and its progression. Free radicals’ involvement in various steps of carcinogenesis is well documented. Reactive oxygen and nitrogen species and their pathways could facilitate cancer development by damaging macromolecules such as DNA or other bio-molecules [3].

Chemotherapeutic drugs are still considered to be the most essential therapeutic approaches for cancer. The problem of drug resistance and toxicity is however a major obstacle in chemotherapeutic treatment. Therefore, it is essential to develop new therapeutic drugs without drug resistance/drug induced toxicity that will be more efficient or will synergize with the existing ones.

Heterocycles are ubiquitous in natural products, pharmaceuticals, organic materials and numerous functional molecules. Therefore, the interest for developing new, versatile and efficient synthesis of heterocycles has always been a thread in the organic and medicinal chemistry [4,5]. Bisindolylmethanes are an important class of nitrogen heterocyles that are known to posses various physiological and pharmacological properties [6] such as antimicrobial [7,8], anti-inflammatory [9], antioxidant activity [10], inhibition of various types of cancer cell growth through induction of apoptosis and its metastasis [11] and glass-formation of high-triplet energy materials [12]. The oxidized form of bisindolylmethanes are utilized as dyes [13] as well as colorimetric sensors [14,15]. Due to the unique biological profiles of bisindolylmethanes, there has been growing interest in the development of a number of efficient synthetic protocols for the preparation of these molecules [16–21]. The most widely used approach to synthesize bisindolylmethanes involves a one-pot reaction of indole with various aromatic aldehydes in the presence of a number of catalysts like Bronsted [22], Lewis acids such as LiClO_4_ [23], In(OTf)_3_ [24], Dy(OTf)_3_ [20], Sc(OTf)_3_ [25,26], CAN [27], ZrOCl_2_ [28], InCl_3_ [29]; heteropolyacids [30]; Ionicliquids [31]; surfactants [32]. The present method for the synthesis of bisindolylmethanes in poly (ethylene glycol) (PEG) supported dichlorophosphate (PEG-P(O)Cl_2_) overcomes these difficulties. From the viewpoint of green chemistry, PEG is an interesting solvent system. PEG has many advantages such as thermal stability and recoverability; it is environmentally benign and a biologically acceptable medium for drug delivery not only as a dehydrating reagent but also acting as a solvent [33].

## 2. Results and Discussion

### 2.1. Chemistry

**Scheme 1:**
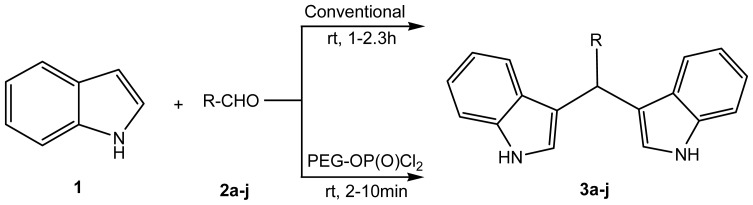
Synthesis of bisindolylmethanes (**3a**–**j**).

In continuation of our research and interest in the development of novel synthetic methodologies [34], herein we report an efficient method for the synthesis of bisindolylmethanes catalyzed by PEG-OPOCl_2_ (Scheme 1). To date, it is the first feasible method for electrophilic substitution at 3-position of indole with various aryl/heteroaryl aldehydes in PEG-OP(O)Cl_2_ medium. It has great advantages including simple and mild experimental conditions, less reaction time, cost-effectiveness due to recyclability of PEG-OP(O)Cl_2_ and applicability to various substituted aryl, and heterocyclic aldehyde substrates. It is an innovative and efficient approach to synthesize bisindolylmethanes while avoiding hazardous solvents.

The main aim for the synthesis of these compounds is to study the variations in cytotoxicity as a function of the chemical structures (Table 1).

To optimize for experimental conditions for the preparation of **3a**–**j**, a typical reaction with Indole (**1**) and Aromatic aldehyde (**2**) was carried out both by both conventional, as well as by PEG-OP(O)Cl_2_ catalyzed, conditions in solvent free method. Product formation in solvent free system of the conventional method has given only 54% in 1–2 h, while with PEG-OP(O)Cl_2_ has given 95% of yield in maximum of 10 min (Scheme 1). The comparative experimental data for the preparation of all the title compounds with conventional, and PEG-OP(O)Cl_2_ catalyzed, pathways in solvent free method are shown in (Table 1).

To determine the appropriate concentration of the catalyst PEG-OP(O)Cl_2_, we investigated the model reaction at different concentrations of catalyst, *i.e.*, 0.1, 0.2, 0.5 and 1 mol%. The product yielded from different concentrations was 72%, 86% and 96% respectively. It was observed that the product yield remained constant at 96% when concentration of the catalyst was increased from 0.5 to 1 mol%. This indicates that 0.5 mol% of PEG-OP(O)Cl_2_ is sufficient for the best result considering the reaction time and yield of product. The results are summarized in (Table 2).

**Scheme 2:**
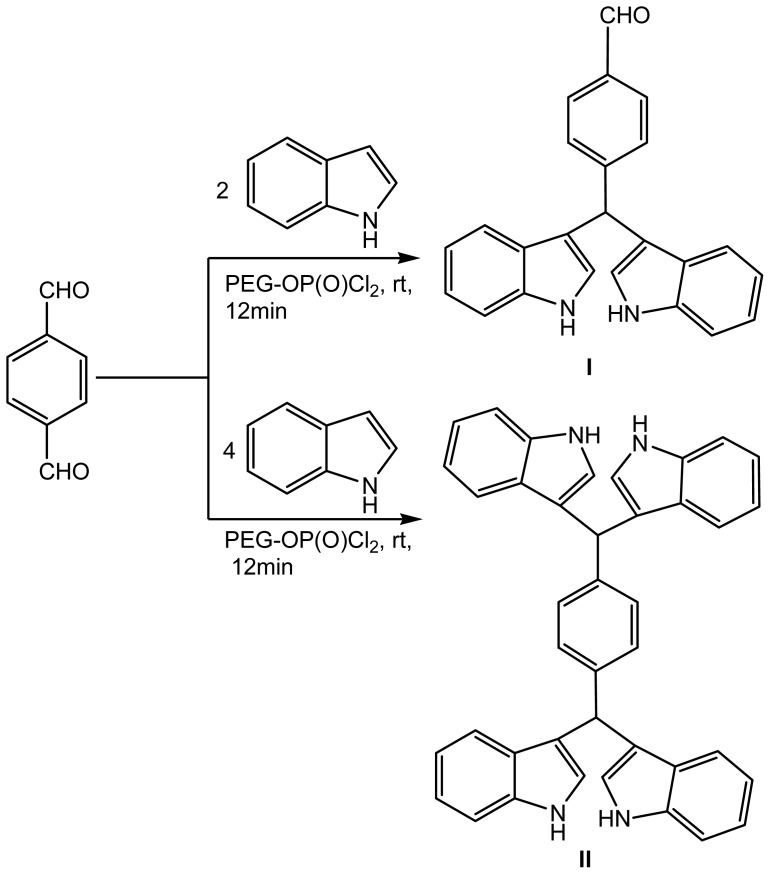
Synthesis of bis(indolyl)methane benzaldehyde (**I**) and di(bis-indolylmethane)benzene (**II**).

Interestingly, the catalyst was effectively used for the synthesis of di-bis(indolyl)methanes from indole and terephthalaldehyde (Scheme 2). The reaction of 2 equivalents of indole with 1 equivalent of terephthalaldehyde proceeded successfully to give bis(indolyl)methane benzaldehyde (**I**) with an excellent yield. On the other hand, using 4 equivalents of indoles to form indoledi(bis-indolylmethane)benzene (**II**) resulted in a high yield within 10–15 min under similar reaction conditions.

The chemical structures of (**3a**–**j**) were confirmed by ^1^H, ^13^C NMR and mass spectral data. The –NH protons gave a singlet in the region of (10.78–8.01) and the methyleneoxy protons resonated as multiplets at (4.06–4.01) [35]. The alkenes protons gave a singlet in the region of 6.73–6.31. Similarly the Ar-CH– protons also gave a singlet in the region of 5.89–5.14.

### 2.2. Biology

In order to investigate the effectiveness of the anticancer properties of synthesized compounds, they were subjected to the well known cytotoxic assay against colon, breast and cervical cancer cell lines, respectively. Concentrations from 25 to 150 μM/mL showed dose dependent anticancer activity after 24 h incubation. The results were presented as the inhibitory concentration that inhibits the growth of cells by 50% (IC_50_) when compared with untreated cells. As shown in Table 3, compound **3a** showed a good anticancer activity with three kinds of cancer cells. Based on these results, compound **3a** has stronger anticancer activity against colon and breast cancer cells.

## 3. Experimental Section

### 3.1. General

Indole, carbonyl compounds, PEG-6000 and POCl_3_ were purchased from Fluka Chemical Company. The progress of the reactions was monitored by thin layer chromatography (TLC) using silica gel 60 F_254_ (pre-coated aluminium sheets) from Merck. ^1^H and ^13^C-NMR spectra were obtained in CDCl_3_ on a Varian 500 MHz NMR spectrometer by using TMS as an internal standard. Infrared Spectra (cm^−1^) were recorded with KBr pellets on a Perkin-Elmer, FT-IR 100 spectrophotometer. E.S.I. mass spectra were recorded on API-3000 mass spectrometer.

### 3.2. Preparation of PEG-POCl_2_ Catalyst

A catalyst was prepared according to reported procedure [34]. To a solution of POCl_3_ (0.93 mL, 10 mmol) in 20 mL of CH_2_Cl_2_, the mixture of PEG-6000 (30 g, 10 mmol OH) and Et_3_N (1.67 mL, 12 mmol) in 80 mL of CH_2_Cl_2_ was added drop-wise at 0–5 °C. Then the resulting solution was stirred for 2 h at room temperature and refluxed for an additional 5 h. The insoluble solid was removed by filtration and the solution was concentrated to a third of its volume. Then appropriate ether was added and the precipitate was filtered to afford a white solid product.

### 3.3. General Procedure for the Synthesis of Bisindolylmethanes (**3a**–**j)**

#### 3.3.1. Method A: Conventional

To 1 mmol of Indole, 2 mmol of carbonyl compound was added and stirred at room temperature. The progress of the reaction was monitored by running TLC (silica gel) using hexane and ethyl acetate (1:3) as a mobile phase at different time intervals. After the completion of reaction, the solvent was removed in a rota-evaporator at reduced pressure and the crude product obtained was purified by column chromatography on silica gel (60–120 mesh), using hexane and ethyl acetate as an eluent to afford the pure compound (**3a**) yield, 55%.

#### 3.3.2. Method B: Catalytic Condition

To a mixture of Indole (1 mmol) and carbonyl compound (2 mmol), PEG-POCl_2_ (0.5 mol%) was added and the resulting mixture was stirred at room temperature. The progress of the reaction was monitored by TLC. After completion of the reaction, the reaction mixture was poured into distil water and stirred for 5 min. The crude product was collected by filtration under suction, washed water and recrystallized from hot ethanol to afford pure bisindolylmethane derivatives.

The physical and spectral data of known compounds (**3a**–**e**, **3i** and **3j**) were found to be in agreement with the reported data [32], and the characterization data of newly synthesized products (**3f**, **3g** and **3h**) are given below.

### 3.4. Cell Lines and Culture

MCF-7, human breast cancer cell line; HT-29, human colon carcinoma cell line and Hela, cervical cancer cell line were obtained from American Type Culture Collection (ATCC, Rockville, MD, USA) and were grown as monolayers in RPMI 1640 medium (GIBCO BRL, Grand Island, NY, USA). Cells were further supplemented with 10% heat-inactivated fetal bovine serum (FBS; Gibco, Auckland, NZ, USA), 100 units/mL penicillin, 100 units/mL streptomycin, at 37 °C, 95% relative humidity, with 5% CO_2_.

### 3.5. *In Vitro* Cytotoxicity Assay

Cell cytotoxicity was measured using the MTT assay. Cancer cells in the exponential growth phase were cultured at a density of 1 × 10^5^ cells/well in 96-well plates. After treatment with various concentration of synthesized bis(indolyl)methanes for 24 h, MTT solution (5.0 mg/mL in phosphate-buffered saline) was added (10 μL/well), and the plates were incubated for another 4 h at 37 °C. Purple formazan crystals were dissolved in 100 μL DMSO per well. After 10 min, the reaction products were colorimetrically quantified at 570 nm with subtraction of the background absorbance at 630 nm by scanning with a microplate reader. Cell viability was determined as a ratio of treated cells to untreated cells (control at 0 μM). A stock solution of compound was prepared in DMSO, and the final concentration of this solvent was kept constant at 0.1%. The control group received DMSO alone. The IC_50_ (the concentration of each substance required for 50% inhibition of tumor activity) was calculated according to standard method. All experiments were performed in triplicate.

3,3′-(phenylmethylene)bis(1H-indole) (**3a**). Solid, mp 124–126 °C; IR (KBr, cm^−1^): 3415 (NH), 3055, 1620, 1600, 1455, 1095, 750; ^1^H NMR (500 MHz, CDCl_3_): δ 7.83 (br s, 2H, NH), 7.36–6.98 (m, 13H, Ar-H), 5.87 (s, 2H), 5.47 (s, IH, Ar-CH); ^13^C NMR (125 MHz, CDCl_3_): 143.9, 136.6, 128.6, 128.1, 127.0, 126.1, 123.5, 121.8, 119.8, 119.6, 119.1, 110.9, 40.1; EIMS: *m*/*z*: 323 [M + H]; Anal. Calc. for C_23_H_18_N_2_: C, 85.7; H, 5.6; N, 8.7%; Found: C, 85.8; H, 5.5; N, 8.6%.

3,3′-((4-nitrophenyl)methylene)bis(1H-indole) (**3b**). Solid; mp 220–222 °C; IR (KBr, cm^−1^): 3420 (NH), 3050, 1595, 1510, 1455, 1340; ^1^H NMR (500 MHz, CDCl_3_): δ 8.12 (d, 2H, *J* = 8.8 Hz), 8.02 (brs, 2H, NH), 7.50 (d, 2H, *J* = 8.8 Hz), 7.40–7.00 (m, 8H, Ar-H), 6.70 (s, 2H), 5.98 (s, IH, Ar-CH); ^13^C NMR (125 MHz, CDCl_3_): 148.4, 146.3, 136.7, 134.8, 129.1, 126.6, 126.1, 123.7, 123.6, 122.3, 121.5, 119.6, 40.0; EIMS: *m*/*z*: 368 [M + H]; Anal. Calc. for C_23_H_17_N_3_O_2_ (367.40): C, 75.2; H, 4.7; N, 11.4%; Found: C, 75.3; H, 4.5; N, 11.6%.

3,3′-((4-chlorophenyl)methylene)bis(1H-indole) (**3c**). Solid; mp 104–106 °C; IR (KBr, cm^−1^): 3415 (NH), 3055, 1490, 1450, 1090; ^1^H NMR (500 MHz, CDCl_3_): δ 7.95 (br s, 2H, NH), 7.35–7.25 (m, 8H, Ar-H), 7.15 (d, 2H, *J* = 7.9 Hz), 7.05 (d, 2H, *J* = 8.3 Hz), 6.65 (s, 2H), 5.80 (s, IH, Ar-CH).

3,3′-(p-tolylmethylene)bis(1H-indole) (**3d**). Solid; mp 96–98 °C; IR (KBr, cm^−1^): 3415 (NH), 3040, 2930, 1610, 1515, 1220, 1055, 775; ^1^H NMR (500 MHz, CDCl_3_): δ 7.98 (brs, 2H, NH), 7.29–7.25 (m, 8H, Ar-H), 7.1 (d, 2H, *J* = 7.6 Hz), 7.05 (d, 2H, *J* = 7.2 Hz), 6.70 (s, 2H), 5.85 (s, 1H, Ar-CH), 2.35 (s, 3H, Ar-CH_3_); EIMS: *m*/*z*: 336; Anal. Calc. for C_24_H_20_N_2_: C, 85.7; H, 6.0; N, 8.3%; Found: C, 85.4; H, 5.9; N, 8.0%.

3,3′-((4-hydroxyphenyl)methylene)bis(1H-indole) (**3e**). Solid; mp 210–211 °C; IR (KBr, cm^−1^): 3350 (OH), 3210 (NH), 3053, 1617, 1601, 1452, 1093, 751; ^1^H NMR (500 MHz, CDCl_3_): δ 8.01 (s, 1H, OH), 7.70 (brs, 2H, NH), 7.38–6.80 (m, 11H, Ar-H), 5.91 (s, 2H), 5.29 (s, IH, Ar-CH).

3,3′-((2,4-dihydroxyphenyl)methylene)bis(1H-indole) (**3f**). Solid; mp 196–198 °C; IR (KBr, cm^−1^): 3450 (–OH), 3275 (–NH), 3053,1617, 1601, 1452, 1093, 751; ^1^H NMR (500 MHz, CDCl_3_): δ 8.01 (s, 2H, OH), 7.78 (brs, 2H, NH), 7.36–6.9 (m, 11H, Ar-H), 5.91 (s, 2H), 5.29 (s, 1H, Ar-CH); ^13^C NMR (125 MHz, CDCl_3_): 164.4, 155.5, 123.5, 122.3, 121.4, 119.9, 119.6, 119.5, 119.2, 118.7, 111.2, 110.7, 103.8, 45.3; EIMS: *m*/*z*: 354; Anal. Calc. for C_23_H_18_N_2_O_2_: C, 77.9; H, 5.1; N, 7.9%; Found: C, 77.6; H, 4.9; N, 7.7%.

3,3′-((3,4-dihydroxyphenyl)methylene)bis(1H-indole) (**3g**). Solid; mp 204–206 °C; IR (KBr, cm^−1^): 3495 (–OH), 3185 (–NH), 3045,1629, 1598, 1444, 1095, 750; ^1^H NMR (500 MHz, CDCl_3_): δ 8.04 (s, 2H, OH), 7.83 (br s, 2H, NH), 7.36–6.98 (m, 11H, Ar-H), 5.87 (s, 2H), 5.27 (s, IH, Ar-CH); ^13^C NMR (125 MHz, CDCl_3_): ^13^C NMR (125 MHz, CDCl_3_): 164.4, 155.5, 136.6, 127.0, 123.4, 121.9, 119.9, 119.8, 119.2, 115.8, 115.1, 110.7, 102.8, 39.4; EIMS: *m*/*z*: 354; Anal. Calc. for C_23_H_18_N_2_O_2_: C, 77.9; H, 5.1; N, 7.9%; Found: C, 77.6; H, 4.9; N, 7.7%.

3,3′-((2,5-dimethoxyphenyl)methylene)bis(1H-indole) (**3h**). Solid; mp 194–196 °C; IR (KBr, cm^−1^): 3410 (NH), 3035,1626, 1610, 1459, 1093, 752; ^1^H NMR (500 MHz, CDCl_3_): δ 7.86 (br s, 2H, NH), 7.31–6.64 (m, 6H, Ar-H), 6.32 (s, 2H), 5.27 (s, IH, Ar-CH), 3.77 (s, 3H, OCH_3_), 3.62 (s, 3H, OCH_3_); ^13^C NMR (125 MHz, CDCl_3_): 153.4, 151.3, 136.6, 134.1, 127.2, 123.4, 121.7, 119.9, 119.4, 119.0, 116.6, 111.7, 110.8, 110.6, 56.5, 55.4, 32.2; EIMS: *m*/*z*: 383; Anal. Calc. for C_25_H_22_N_2_O_2_: C, 78.5; H, 5.8; N, 7.3%; Found: C, 78.2; H, 5.5; N, 6.8%.

3,3′-((4-benzykoxy)phenylmethylene)bis(1H-indole) (**3i**). Solid; mp 264–266 °C; ^1^H NMR (400 MHz, DMSO-*d*_6_): δ 10.78 (brs, 2H, NH), 7.37–6.77 (m, 19H, Ar-H), 6.31 (s, 2H, Ar-H), 5.14 (s, 1H, Ar-CH), 4.06–4.01 (m, 2H, O-CH_2_); ^13^C NMR (100 MHz, DMSO*d*_6_): δ 155.7, 137.8, 137.0, 133.7, 129.5, 128.6, 127.9, 127.5, 127.4, 121.2, 120.7, 119.3, 118.5, 118.2, 112.8, 111.8, 69.7, 47.8; EIMS: *m*/*z*: 428 [M]^+^.

3,3′-((2-pyridylphenyl)methylene)bis(1H-indole) (**3j**). Solid; mp 264–266 °C; ^1^H NMR (400 MHz, DMSO-*d*_6_): δ 10.78 (brs, 2H, NH), 8.36 (1H, d, *J* = 7.8 Hz), 7.37–6.77 (m, 15H, Ar-H), 6.31 (s, 2H, Ar-H), 5.14 (s, 1H, Ar-CH); ^13^C NMR (100 MHz, DMSO *d*_6_): *δ* 154.6, 147.2, 137.4, 136.0, 135.7, 133.5, 128.6, 127.9, 127.5, 127.2, 124.1, 121.2, 120.7, 119.3, 118.5, 118.2, 112.8, 111.8, 69.7; EIMS: *m*/*z*: 400 [M + H]^+^.

## 4. Conclusions

In conclusion, a simple, efficient, green, and eco-friendly procedure is described in this study for the synthesis of bis(indolyl)methanes in the presence of PEG-OP(O)Cl_2_. This protocol provides a low cost procedure for the synthesis of these compounds and also evaluated their anticancer activity.

## Figures and Tables

**Table 1:** Synthesis of bis(indolyl)methanes (**3a**–**j**) with conventional and PEG-OP(O)Cl_2_ catalyzed methods.

Compound	R	Conventional method	PEG-POCl_2_ method	Melting point	
		Time (h)/Yield (%)	Time (min)/Yield (%)	Found	Literature
**3a**	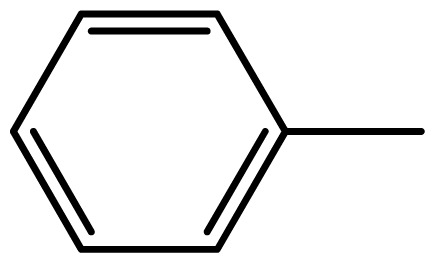	2/55	3/95	124–126	125–127
**3b**	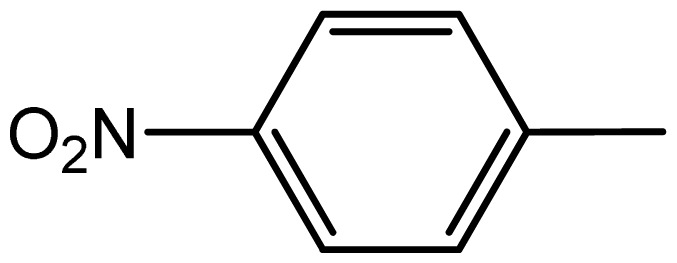	1.2/65	5.2/92	220–222	221–223
**3c**	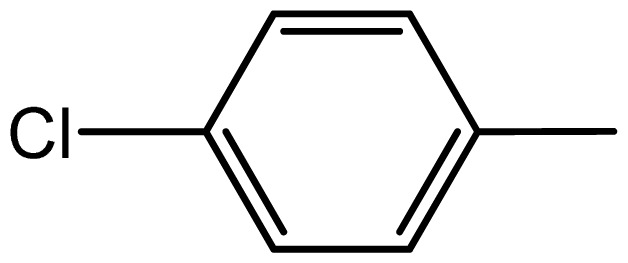	1.3/68	5.5/94	104–106	102–104
**3d**	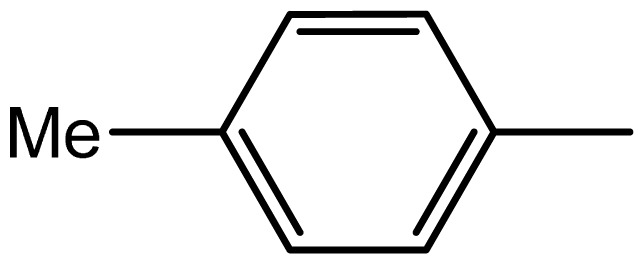	1.6/63	6/94	96–98	97–99
**3e**	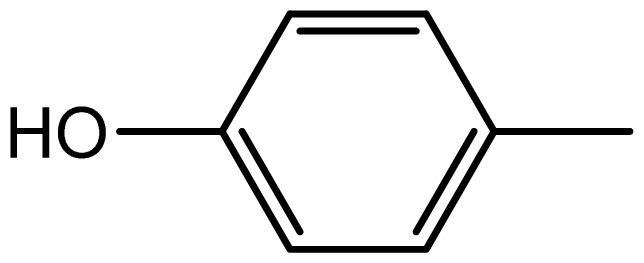	1.6/63	6/94	122–124	122–124
**3f**	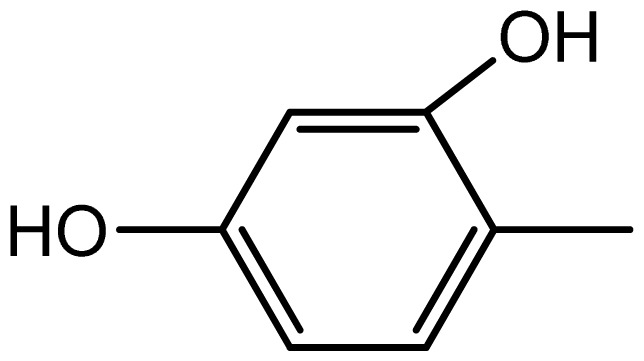	2.2/65	8/95	196–198	-
**3g**	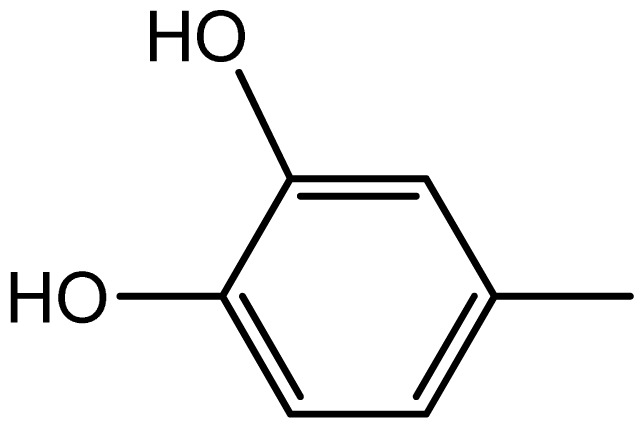	2.2/64	8/94	204–206	-
**3h**	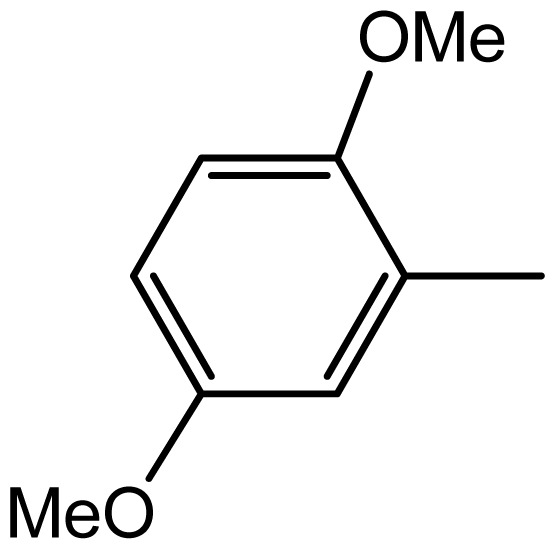	2/75	4/96	194–196	-
**3i**	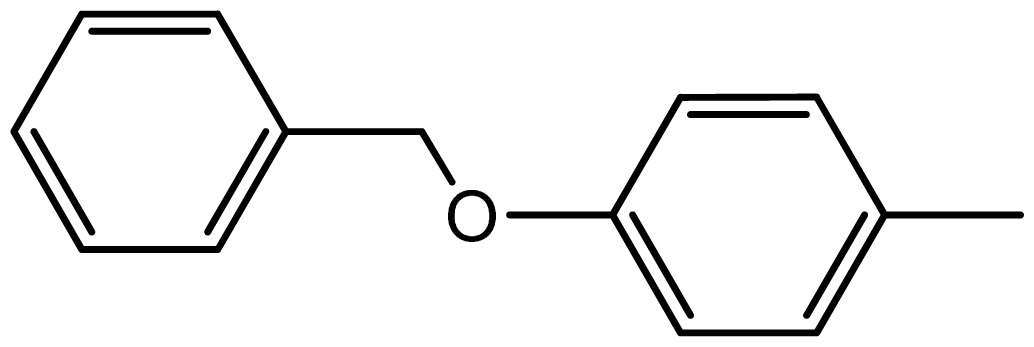	2.3/67	10/95	264–266	264–266
**3j**	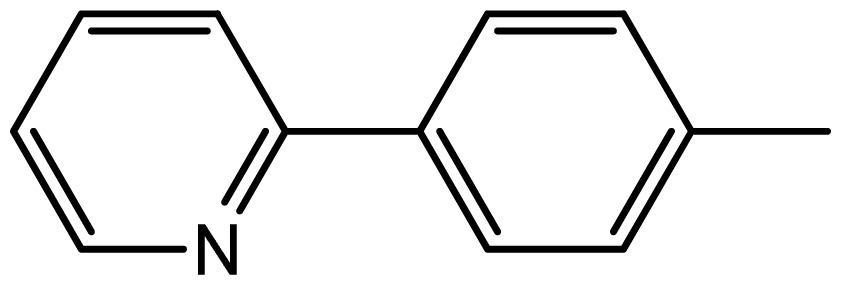	1.9/66	9/94	180–182	182–183

**Table 2:** Optimization of catalyst at different concentrations for the preparation of **3a**.

Entry	Catalyst (Mole %)	Yield of the product
1	0.1	72
2	0.2	86
3	0.5	95
4	1	95

**Reaction Conditions**: 1. Mole (%) is 0.1, Reaction time 10 min, RoomTemperature; 2. Mole (%) is 0.2, Reaction time 5 min, Room Temperature; 3. Mole (%) is 0.5, Reaction time 3 min, Room Temperature.

**Table 3:** Cytotoxic activity of synthesized compounds (IC_50_ concentration).

S.No.	Compound	Hela cells (μM)	HT 29 (μM)	MCF-7 (μM)
1	**3a**	86.3	62.3	138
2	**3b**	>150	>150	>150
3	**3c**	>150	>150	136
4	**3d**	>150	91.7	>150
5	**3e**	>150	>150	>150
6	**3f**	95.4	128.3	145.8
7	**3g**	>150	>150	>150
8	**3h**	>150	142.7	149.3
9	**3i**	129.5	98.4	>150
10	**3j**	139.3	89.7	>150
